# Using Synthetic Biology to Distinguish and Overcome Regulatory and Functional Barriers Related to Nitrogen Fixation

**DOI:** 10.1371/journal.pone.0068677

**Published:** 2013-07-25

**Authors:** Xia Wang, Jian-Guo Yang, Li Chen, Ji-Long Wang, Qi Cheng, Ray Dixon, Yi-Ping Wang

**Affiliations:** 1 State Key Laboratory of Protein and Plant Gene Research, College of Life Sciences, Peking University, Beijing, China; 2 Biotechnology Research Institute, Chinese Academy of Agriculture Science, Beijing, China; 3 Department of Molecular Microbiology, John Innes Centre, Norwich Research Park, Norwich, United Kingdom; Niels Bohr Institute, Denmark

## Abstract

Biological nitrogen fixation is a complex process requiring multiple genes working in concert. To date, the *Klebsiella pneumoniae nif* gene cluster, divided into seven operons, is one of the most studied systems. Its nitrogen fixation capacity is subject to complex cascade regulation and physiological limitations. In this report, the entire *K. pneumoniae nif* gene cluster was reassembled as operon-based BioBrick parts in *Escherichia coli*. It provided ∼100% activity of native *K. pneumoniae* system. Based on the expression levels of these BioBrick parts, a T7 RNA polymerase–LacI expression system was used to replace the σ^54^-dependent promoters located upstream of *nif* operons. Expression patterns of *nif* operons were critical for the maximum activity of the recombinant system. By mimicking these expression levels with variable-strength T7-dependent promoters, ∼42% of the nitrogenase activity of the σ^54^-dependent *nif* system was achieved in *E. coli*. When the newly constructed T7-dependent *nif* system was challenged with different genetic and physiological conditions, it bypassed the original complex regulatory circuits, with minor physiological limitations. Therefore, we have successfully replaced the *nif* regulatory elements with a simple expression system that may provide the first step for further research of introducing *nif* genes into eukaryotic organelles, which has considerable potentials in agro-biotechnology.

## Introduction

Nitrogen fixation is a pivotal process in global nitrogen cycling and is of huge ecological and agronomic importance. The ability to fix nitrogen is distributed in bacteria and archaea [1]. Among these organisms, the free-living diazotroph *Klebsiella pneumoniae* has been extensively studied at the genetic level. A cluster of 21 genes organized into seven operons is required for the biosynthesis, activity, and regulation of nitrogenase, a complex enzyme consisting of two component metalloproteins. The process of dinitrogen reduction is stringently controlled in this organism, and *nif* gene transcription is regulated by a cascade system [1]. The first level of regulation contains the two-component NtrB-NtrC regulatory system, which provides global control in response to the nitrogen source and modulates the expression of the *nifLA* operon. Under nitrogen-limiting conditions, NtrC is phosphorylated and activates transcription of the *nifLA* operon. In the second tier of regulation, the *nifLA* gene products then control expression of the remaining *nif* operons. NifL regulates the activity of NifA in response to both nitrogen and oxygen [2]. NifA, together with the Integration Host Factor (IHF) and the σ^54^-holoenzyme form of RNA polymerase (σ^54^), initiates transcription at the other *nif* promoters [3], [4].

One of the fundamental aims of synthetic biology is to design regulatory and metabolic pathways that can be readily introduced into different biological systems to provide novel functions. An important consideration in the synthetic design is to achieve balanced levels of gene expression in order to provide the appropriate stoichiometry of molecular components. Quantitative and synthetic biology (QSB) is a powerful biotechnological tool that uses quantitative analysis and engineering approaches to manipulate biological systems to obtain the balanced expression of multiple genes. In prokaryotes, gene expression is mainly controlled at the transcriptional level, and the promoter is the most manipulatable element [5]. Hence, promoter replacement is commonly used to modify the genetic regulation of a given gene or gene cluster [6].

In the 1970s, the *K. pneumoniae nif* gene cluster was transferred into *Escherichia coli* thus creating the first engineered diazotroph [7]. Subsequently, a broad host range plasmid (pRD1) carrying the complete cluster was constructed [8]. Further exploitation of this cluster for biotechnological purposes requires synthetic biology tools to remove the complex native regulatory system and replace the promoters to provide a more “universal” expression system. However, redesigning the *nif* cluster in this way is complicated by the number of gene products involved and the complex nitrogenase assembly pathway, which involves the biosynthesis of unique metalloclusters. Furthermore, as the ratios of the *nif*-encoded proteins are important for both nitrogenase biosynthesis [9] and activity [10], it is important to balance the expression of individual operons to ensure that appropriate protein stoichiometry is obtained. Therefore, it is necessary to “mimic” the expression levels in the native system to achieve a functionally active enzyme.

Here we used the T7 RNA polymerase transcription system for the expression of *nif* genes to determine whether the recombinant genes could work independently of the native regulatory factors. T7 RNA polymerase is a single ∼100-kDa polypeptide that is widely used for gene expression in both prokaryotes and eukaryotes [11], [12], [13]. This enzyme initiates transcription from a conserved promoter sequence spanning from –17 to +6, and the relative strength of single base-pair variants in each residue has been characterized [14]. To balance the expression of different *nif* operons, the *nif* promoters were replaced with T7 promoter variants according to required promoter strengths. The *lac* operator was used to regulate the T7 promoters so that *nif* gene expression was responsive only to the small molecular inducer isopropyl-β-thiogalactoside (IPTG). Finally, we reassembled the recombinant *nif* genes to generate an active cluster that provided a high level of nitrogenase activity. Replacing the optimum T7 promoter with other T7 promoter variants resulted in a lower level of nitrogenase activity, confirming that coordinated and balanced expression of the *nif* gene cluster was essential for maximum activity. After induction, the recombinant system bypasses the native regulatory networks and some of the intrinsic physiological limitations.

## Materials and Methods

### Bacterial strains and plasmids

Bacterial strains and plasmids used in this study are listed in Table 1. The *rpoN::kan*, *ntrBC::kan* mutant alleles were moved into strain JM109 by P1 transduction. The *himA::kan* and *himD::Tet* mutations were constructed by a one-step method for gene inactivation in *E. coli* through λ Red recombination system [15]. We used PCR to confirm the mutated regions after mutants were generated, and the PCR products were sequenced to verify.

**Table 1:** Bacterial strains and plasmids used in this work.

Strains/Plasmids	Relevant characteristics	Reference or source
***K. pneumonia***		
M5a1	wild type	Lab stock
UNF921	Δ(*his-nif*), *lacZ::nifH*, *recA*, *rsdR*	[8]
***E. coli***		
DH5α	F−, φ80d, *lacZΔM15*, Δ(*lacZYA-argF*), U169, *deoR*, *recA1*, *endA*, *hsdR17 (rk−, mk+)*, *phoA*, *supE44*, *gyrA96*, *relA1*	Takara
BL21(DE3)	F−, *ompT*, *hsdSB (rB− mB−)*, *gal*, dcm (DE3)	Takara
JM109	*recA*, *endA1*, *gyrA96*, *hsdR17*, *supE44*, *relA1*, Δ(*lac-proAB*)/F' [*traD36*, *proAB+*, *lacIq*, *lacZΔM15*]	Takara
VH1000T	Strain for β-galactosidase activity assay, Tet^R^	Lab stock
ΔhimA	Deletion derivative of E. coli JM109; *himA::kan*	This study
ΔhimD	Deletion derivative of E. coli JM109; *himD::Tet*	This study
ΔrpoN	Deletion derivative of E. coli JM109; *rpoN::kan*	This study
ΔntrBC	Deletion derivative of E. coli JM109; *ntrBC::kan*	This study
**Plasmids**		
pRD1	P-group R factor, *nif+*, *his*+, Km^R^, Cb^R^, Tc^R^	[8]
pUC18	ColE1, *lacZ'*, Ap^R^	[31]
pBluescript II SK (+)	ColE1, *lacZ'*, Ap^R^	Stratagene
pBR322	pMB1, Ap^R^	[32]
pACYC184	p15A, Cm^R^	[33]
pST1021	pACYC184 derivative, expresses nifA constitutively, Cm^R^	Lab stock
pET28a	Expression vector, Km^R^	Novagen
pET28a-M5	pET28a derivative, in which P_T7WT_ was replaced with P_T7M5_, Km^R^	This study
pET28a-M6	pET28a derivative, in which P_T7WT_ was replaced with P_T7M6_, Km^R^	This study
pKU7017	pACYC184 derivative carrying 7 *nif* operons, Cm^R^	This study
pKU7180	pACYC184 derivative carrying 6 T7-dependent *nif* operons(P_T7WT_::*nifHDKTY*,P_T7WT_::*nifJ*,P_T7M5_::*nifENX*,P_T7M5_::*nifBQ*,P_T7M6_::*nifUSVWZM*,P_T7M6_::*nifF*) and and *lacIq*, Cm^R^	This study
pKU7181	pKU7180 derivative carrying *nifLA* operon driven by its original promoter, Cm^R^	This study
pKU7380	pKU7180 derivative carrying *nifLA* operon driven by the T7 promoter, Cm^R^	This study
pKU7093	pBR322::*T7 RNAP*, Ap^R^	This study
pKU7450	P_Tet_::*T7 RNAP* cassette was cloned into pBluescript II SK (+), Ap^R^	This study
pRW50	PSC101, lac reporter vector, Tc^R^	[34]
pRWX1	pRW50 derivative carrying a kanamycin resistance cassette, Km^R^	Lab stock
pRWX2	pRW50 derivative, in which the segment of *E. coli trp* operon was deleted, and contained the original ribosome binding site upstream of *lacz* gene, Km^R^	This study
pRWX2- *nifBQ*p	*nifBQ*p::*lacZ* fusion in pRWX2, Km^R^	This study
pRWX2- *nifEN*Xp	*nifENX*p::*lacZ* fusion in pRWX2, Km^R^	This study
pRWX2- *nifHDKTY*p	*nifHDKTY*p::*lacZ* fusion in pRWX2, Km^R^	This study
pRWX2-*nifUSVWZM*p	*nifUSVWZM*p::*lacZ* fusion in pRWX2, Km^R^	This study
pRWX2- *nifJ*p	*nifJ*p::*lacZ* fusion in pRWX2, Km^R^	This study
pRWX2- *nifF*p	*nifF*p::*lacZ* fusion in pRWX2, Km^R^	This study

Ap, ampicillin; Cm, chloramphenicol; Km, kanamycin; Tc, tetracycline; R, resistance; *nifBQ*p, *nifBQ* promoter; *nifENX*p, *nifENX* promoter; *nifHDKTY*p, *nifHDKTY* promoter; *nifUSVWZM*p, *nifUSVWZM* promoter; *nifJ*p, *nifJ* promoter; *nifF*p, *nifF* promoter; ▵, deletion; ::, novel joint.

### Growth medium and chemicals

Luria-Bertani (LB) broth and M9 medium for *E. coli* growth were prepared as previously described [16]. The medium for the nitrogenase activity assay contained (per liter) 10.4 g Na_2_HPO_4_, 3.4 g KH_2_PO_4_, 26 mg CaCl_2_·2H_2_0, 30 mg MgSO_4_, 0.3 mg MnSO_4_, 36 mg ferric citrate, 7.6 mg Na_2_MoO_4_·2H_2_0, 10 µg para-aminobenzoic acid, 5 µg biotin, 2% (w/v) glucose, and a nitrogen source as indicated (10 mM glutamate was used as nitrogen source in this study, with the exception of the in experiments where various nitrogen sources were examined). When necessary, 50 µg/ml ampicillin, 25 µg/ml chloramphenicol, 10 µg/ml tetracycline, and 25 µg/ml kanamycin were used.

### Plasmid construction

The rationale of the genetic design is outlined in the **Results**. Plasmid pKU7017 is a pACYC184 derivative containing all seven σ^54^-dependent *nif* operons with BioBrick interfaces. To construct pKU7017, seven *nif* operons were PCR-amplified from plasmid pRD1 [8], and each PCR products was cloned into vector pBluescript II SK (+) and verified by sequencing. The first operon was digested with *Xba*I and *Spe*I and inserted into the *Xba*I site of pACYC184, and then another six operons were assembled onto the plasmid backbone in sequence.

**Figure 1:**
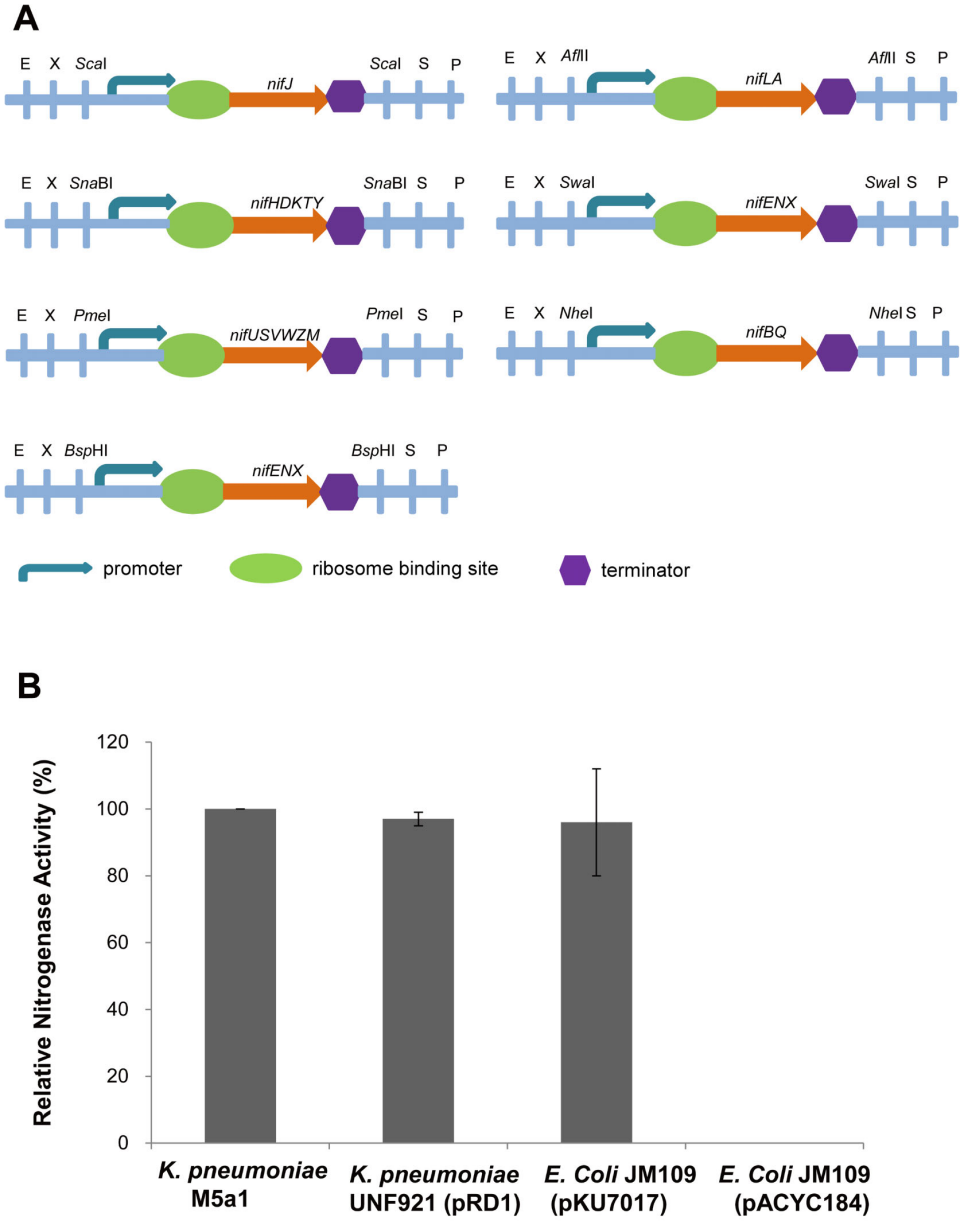
Assembly and functional analysis of the K. *pneumoniae nif* gene cluster in *E. coli*. (**A**) Linear view of the *nif* gene region in the plasmid pKU7017 with the BioBrick interfaces. E, *Eco*RI; X, *Xba*I; S, *Spe*I; P, *Pst*I; (**B**) relative nitrogenase activity of wild-type *K. pneumoniae* M5a1, *K. pneumoniae* UNF921 (pRD1), *E. coli* JM109 (pKU7017), and *E. coli* JM109 (pACYC184). Plasmid pKU7017 refers to the plasmid containing the reconstituted σ^54^-dependent *nif* system and pACYC184 was used as a negative control. Each experiment was repeated at least three times, and the error bars represent standard error.

Plasmid pKU7180 is a pACYC184 derivative carrying six *nif* operons (the *nifLA* operon was not included), in which all *nif* promoters and terminators were replaced with T7 promoter variants and T7 terminators, respectively. Single base-pair substitutions were introduced according to the relative strength of T7 promoter variants [14] and analysis of the β-galactosidase activities of *nif* promoter-*lacZ* fusions. Primers with *Spe*I-*Hin*dIII restriction sites were used to amplify *nif* operons without the promoter and terminator, and then the *Spe*I-*Hin*dIII fragments were inserted into *Xba*I-*Hin*dIII sites of vector pET28a (Novagen). A synonymous mutation was made to delete the *Hin*dIII restriction site in the *nifHDKTY* operon. Because *Xba*I and *Spe*I are isocaudomers, this process created a mixed *Spe*I-*Xba*I junction that could not be cut with either endonuclease and did not influence the subsequent assembly of modulons. The *nif* genes with variant T7 promoters and corresponding T7 terminators were PCR amplified, each operon was also flanked with *Spe*I-*Xba*I restriction sites and a unique restriction site showed in Figure 1A. Finally, the recombinant modulons were assembled into pACYC184 to construct plasmid pKU7180.

Plasmid pKU7093 is a pBR322 derivative containing the T7 RNA polymerase gene (T7 RNAP) under the control of the *tet* promoter. To substitute the tetracycline resistance (*tet*) gene with the T7 RNAP gene on pBR322, we created an *Nco*I restriction site at the translation start site of the *tet* gene, then cloned the T7 RNAP gene into the *Nco*I/*Bam*HI sites of the newly constructed vector. Plasmid pKU7450 is a pBluescript II SK (+) derivative carrying the P_tet_::T7 RNAP cassette. The cassette was cut with *Hin*dIII/*Sac*I from pKU7093 and then cloned into the multiple cloning site of pBluescript II SK (+).

### Assay of β-galactosidase activity

Plsmid pRWX2 were used for the transcriptional fusions of *nif* promoters to the *lacZ* gene. It is a pRW50 derivative, in which the segment of *E. coli trp* operon was deleted, and it contains the original ribosome binding site upstream and complete ORF encoded by *lacZ*. The *nif* promoters were PCR amplified from plasmid pRD1 [8], and then cloned into pRWX2.

β-galactosidase assays were performed according to Miller [17]. The *E. coli* MG1655 *lacZYA* mutant strain VH100T was co-transformed with pST1021 (from which the *K. pneumoniae nifA* gene is constitutively expressed) and the relevant plasmid containing the appropriate *nif* promoter (*nif*p) –*lacZ* fusion. Cells were grown overnight in M9 medium and then diluted into 10 ml fresh M9 medium, and β-galactosidase activities were measured when cells reached logarithmic growth phase.

### Assay of nitrogenase activity

The acetylene reduction method was used to assay the nitrogenase activity as described [18]. To measure nitrogenase activity of the *K. pneumoniae* M5a1, and *E. coli* JM109 (pKU7017) strains, cells were initially grown overnight in M9 medium. For optimal IPTG induction, the JM109 (pKU7450, pKU7180) strain was grown in M9 medium to an OD_600_ of 0.4–0.6. The cells were then diluted into 5 ml nitrogenase activity assay medium in 25 ml sealed tubes (supplemented with appropriate antibiotics and IPTG), and grown to a final OD_600_ of ∼0.4. Air in the tube was repeatedly evacuated and flushed with argon. After incubation at 30°C (or 37°C) for 16–20 hr, 1 ml acetylene was injected, and the gas phase was analysed 3 hr later with a SHIMADZU GC-2014 gas chromatograph. Data presented are mean values based on at least three replicates.

### Western blot

The proteins were applied to a 10% (w/v) SDS/polyacrylamide gel and then analyzed by immunoblotting. The immunoblots were probed with a 1∶1000 dilution of NifH rabbit polyclonal antibody. The antiserum against NifH was a gift from Prof. Jilun Li of China Agriculture University. The antibody-antigen complex was visualized with alkaline phosphatase conjugated to goat anti–rabbit IgG. For western blot analysis, samples were taken just after testing nitrogenase activity, with 20 mg total protein (or supernatant) after sonication loaded for each sample.

## Results

### The *K. pneumoniae nif* gene cluster can be reassembled and functionally expressed in *E. coli*

To facilitate manipulation of the *K. pneumoniae nif* gene cluster, we first followed the BioBrick design principles [19] to flank each of the seven native *nif* operons with restriction sites. Each operon was also flanked with a unique restriction site to facilitate individual module replacement (Figure 1A). When introduced into the multicopy plasmid pACYC184 (designed as pKU7017, see also Table 1) and transformed into *E. coli* strain JM109, the reassembled *nif* cluster exhibited nitrogenase activity as measured by acetylene reduction. The level of activity was 30.2 nmol ethylene/min/mg protein, corresponding to ∼100% of the activity shown by *K. pneumoniae* wild-type strain M5a1 and similar to *K. pneumoniae nifΔ* strain UNF921 carrying the pRD1 *nif* plasmid (Figure 1B).

### A T7 RNA polymerase based transcription system effectively drives *nif* gene expression in *E. coli*

**Figure 2:**
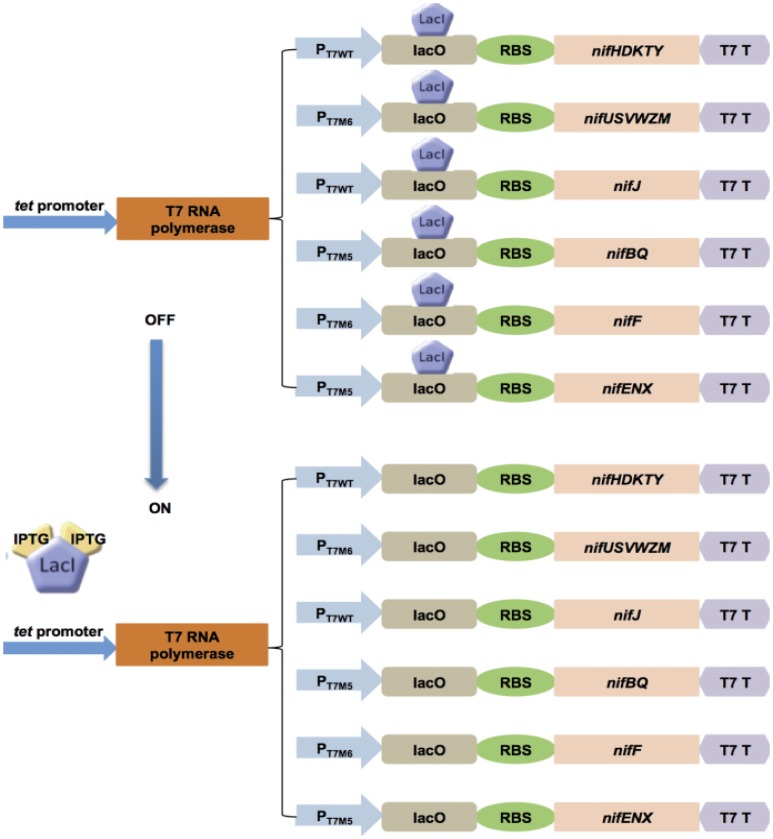
Construction of the nitrogen fixation “expression cassette” with the T7 RNA polymerase based expression system. Top: off state (no induction); LacI represses the transcription of all *nif* genes. Bottom: on state (induced); addition of IPTG turns *nif* gene transcription on by releasing the LacI mediated repression. The T7 RNA polymerase gene is expressed from the constitutive *tet* promoter.

Having shown that the *nif* gene cluster functioned well in *E. coli* when split into BioBrick operon parts, we then constructed an “expression cassette” for nitrogen fixation, in which a T7 RNA polymerase based transcription system drives *nif* gene expression. Native σ^54^-dependent promoters were replaced with T7 promoters and termination signals present in the native operons were replaced by the T7 terminator (Figure 2). Since the ratios of the *nif* encoded proteins are important for both nitrogenase biosynthesis and activity, T7 promoter variants of different strengths were used to replace the different *nif* promoters in order to maintain the appropriate “balance” in the levels of each gene product. To evaluate the relative activities of *nif* promoters, we fused the *lacZ* reporter gene with each of the *nif* promoters (the promoter of the regulatory *nifLA* operon was not included), and measured β-galactosidase activities. Under these conditions, the native *nifJ* and *nifH* promoters exhibited the highest expression levels amongst these σ^54^-dependent promoters, whereas the *nifU* and nifF promoter had the lowest expression level (Table S1.)

Taking into account the above measurements, the six *nif* operon promoters were replaced by optimum-strength T7 promoters. In particular, the wild-type T7 promoter (P_T7WT_) was used to drive the structural genes *nifHDKTY*, which are highly expressed in diazotrophs, as the nitrogenase component proteins can represent up to 10% of total cell protein under nitrogen-fixing conditions [20]. We also used the wild-type T7 promoter to drive *nifJ*, which is also highly expressed. A weak promoter (P_T7M6_, with a G substitution at −4, resulting in 20% of the wild-type T7 promoter activity [14]) was used to control the *nifF* and *nifUSVWZM* operons, whereas the *nifENX* and *nifBQ* operons were controlled by a medium-strength promoter (P_T7M5_, with a G substitution at −2, providing 40% of the wild-type T7 promoter activity [14]). To control gene expression, the *lac* operator was introduced between each T7-derived promoter and the ribosome binding site of the first *nif* gene in each operon. These manipulations resulted in a total of six redesigned modulons, each of which contained a T7-derived promoter with the required strength, a *lac* operator, a *nif* gene/operon, and a T7 terminator (Figure 2). The *lacIq* gene, which controls the *lac* operator, was also introduced together with the six modulons to assemble a pACYC184-based plasmid (pKU7180) containing the redesigned *nif* gene cluster, hereafter referred to as the T7-dependent *nif* expression system. Transcription from the T7 promoters was driven by a separated plasmid (pKU7450), in which T7 RNA polymerase was expressed from the constitutive *tet* promoter (see also Table 1).

When plasmids pKU7180 and pKU7450 were introduced into *E. coli* strain JM109, IPTG-inducible nitrogenase activity was recovered as measured by acetylene reduction. Very low nitrogenase activity was detectable in the absence of IPTG, implying that the Lac repressor effectively repressed transcription of the *nif* operons. Titration of the inducer revealed that 0.2 mM IPTG resulted in the highest nitrogenase activity (12.6 nmol ethylene/min/mg protein; (Table 2)). This corresponds to 41.8% of the activity exhibited by the reconstituted σ^54^-dependent *nif* system (*nif* system assembled as BioBrick parts). Notably, nitrogenase activity decreased at higher IPTG concentrations (Table 2), possibly because of the deleterious overexpression of component proteins. Hence, we used 0.2 mM IPTG for induction in subsequent experiments.

**Table 2:** IPTG controlled nitrogenase activities of *E. coli* JM109 strain carrying the T7-dependent *nif* system.

IPTG (mM)	Relative nitrogenase activity (%)
0	8.3±0.8
0.1	56.7±16.3
0.2	100.0
0.4	63.2±12.2
0.6	51.7±1.2
0.8	43.7±15.7
1	21.8±1.3

Plasmids pKU7180 and pKU7450 was transformed into *E. coli* JM109 strain, and nitrogenase activities are shown as a percentage of the activity when 0.2 mM IPTG was used for induction. Each experiment was repeated at least three times, and the error bars represent standard error.

### Coordinated and balanced expression of *nif* genes is important for nitrogenase activity

**Figure 3:**
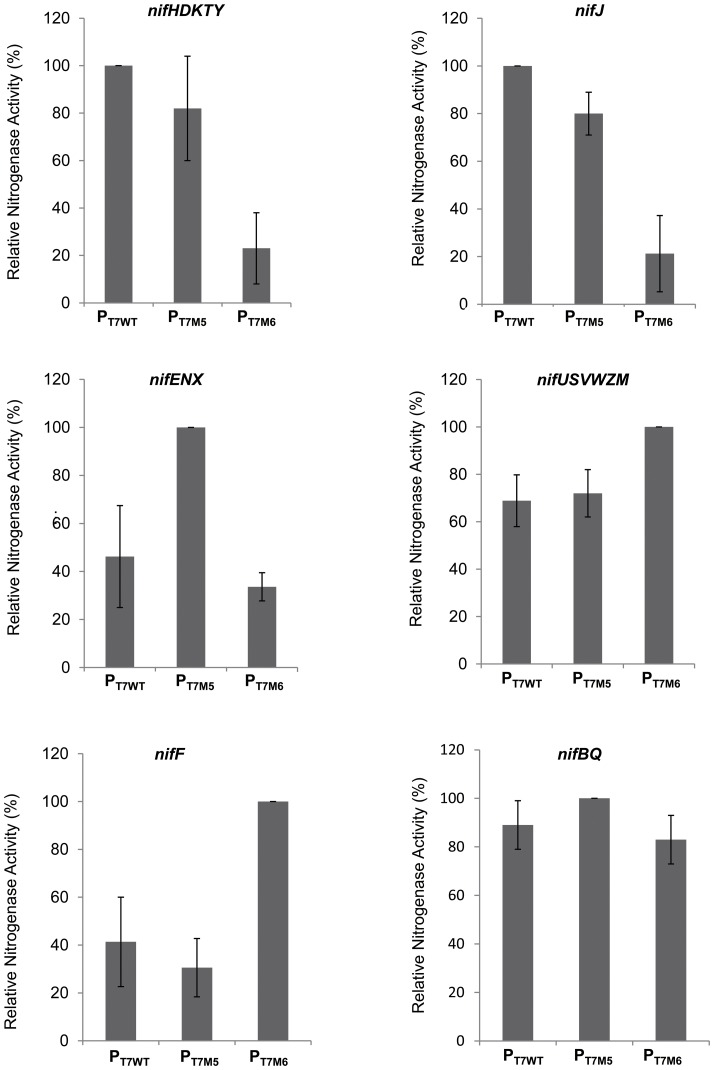
Influence of T7 promoter strength on nitrogenase activity. The optimal T7 promoter for each operon was tested using three different T7 promoters (P_T7WT_, P_T7M5_, and P_T7M6_). Each variant promoter module was introduced as a single substitution into the complete *nif* expression cassette. Nitrogenase activity with the optimal T7 dependent promoter construction (plasmid pKU7180) represents 100% in each case and 0.2 mM IPTG was used for induction. Each experiment was repeated at least three times, and the error bars represent standard error.

To evaluate the robustness of the T7-dependent *nif* expression cassette and, in particular, the importance of relative promoter strengths, each of the six modulons was reconstructed by replacing the optimum T7 promoter with the other two T7 promoter variants, resulting in 12 alternative modulons. For example, P_T7WT_, the *nifHDKTY* modulon, was replaced with either the P_T7M5_ or the P_T7M6_ promoter variants to drive expression of the *nifHDKTY* modulon. When each of the variant modulons was introduced as single substitutions in the complete *nif* expression cassette, most replacements resulted in lower nitrogenase activities (Figure 3). As anticipated, decreasing the expression of the structural genes *nifHDKTY* significantly lowered activity, particularly in the case of the P_T7M6_ variant, which has 20% of the promoter strength of P_T7WT_ [14]. Similar results were obtained with *nifJ*, which in the native *K. pneumoniae nif* system is bidirectionally transcribed with respect to *nifH*, and their σ^54^-dependent promoters share regulatory features. In contrast, high-level expression of *nifF* was deleterious, perhaps because protein overexpression results in covalent modification of the flavodoxin by coenzyme Q, which prevents electron transfer from NifJ to the Fe protein [21]. However, the *nifBQ* operon seems more robust with respect to promoter replacement.

Taken together, these results substantiate our choice of variant T7 promoters in providing mimics of the native system and indicate that the stoichiometry of *nif* gene expression is still very important for nitrogenase assembly and activity in this redesigned expression system.

### The T7-dependent *nif* system bypasses the involvement of native regulatory factors

**Figure 4:**
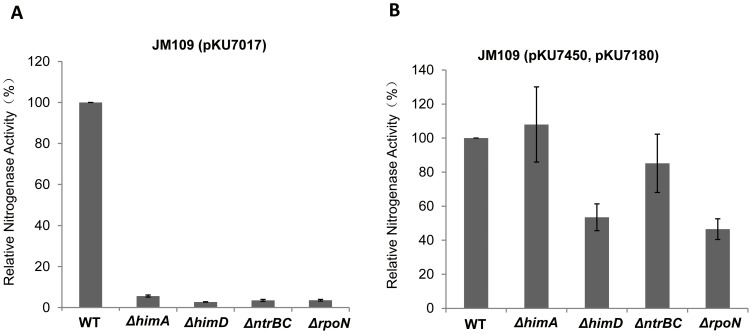
Influence of host regulatory genes on the σ^54^- and T7-dependent *nif* systems. Relative nitrogenase activity of mutant *E. coli* strains with (**A**) the σ^54^-dependent *nif* system and (**B**) the T7-dependent *nif* system. WT indicates the parent strain JM109, and 0.2 mM IPTG was used for induction. Each experiment was repeated at least three times, and the error bars represent standard error.

As mentioned above, expression of the native *nif* gene cluster is subject to complex cascade regulation. Factors include the PII signal transduction proteins encoded by *glnB* and *glnK*, the NtrBC two-component system, the *nif* specific regulatory proteins NifL and NifA, and the requirement for σ^54^ [3], [4]. In addition, Intergration Host Factor (IHF) plays an important role in modulating the activity of σ^54^-dependent promoters [4]. To compare the influence of regulatory and physiological factors in the redesigned *nif* expression cassette with that of the native σ^54^-dependent system, we introduced appropriate plasmids into various *E. coli* mutant strains. As demonstrated previously the native system was completely dependent on the nitrogen regulation genes *ntrBC*, the *rpoN* gene (which encodes σ^54^), and the genes *himA* and *himD*, encoding the α and ß subunits of IHF respectively (Figure 4A). In contrast, the T7-based expression system significantly bypassed the requirement for these factors (Figure 4B). Although some decrease in activity was observed in the *himD* and *rpoN* mutants, we assume that this is an indirect effect that may result from the pleiotropic influence of these mutations on cellular physiology.

### Influence of nitrogen sources on the output of the T7-dependent *nif* system

**Figure 5:**
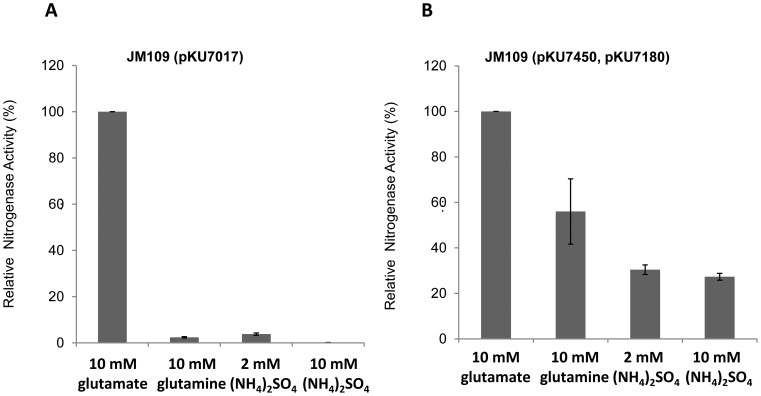
Influence of nitrogen sources on nitrogenase activities of the σ^54^-, and T7-dependent *nif* systems. Relative nitrogenase activity of mutant *E. coli* strains with (**A**) the σ^54^-dependent *nif* system and (**B**) the T7-dependent *nif* system under various nitrogen conditions. Activities were measured in the presence of the different nitrogen sources indicated on the x axis. The nitrogenase activity of cells grown in medium contained 10 mM glutamate as the sole nitrogen source was considered to be 100%, and 0.2 mM IPTG was used for induction. Each experiment was repeated at least three times, and the error bars represent standard error.

In *K. pneumoniae*, *nif* gene expression can be activated only under nitrogen-limiting conditions. Accordingly, the reconstituted σ^54^-dependent *nif* system in *E. coli* showed very little nitrogenase activity when either ammonium (2 or 10 mM) or 10 mM glutamine was present in the medium (Figure 5). As mentioned previously, this is a consequence of the influence of these fixed nitrogen sources on both the NtrBC and NifLA regulatory systems [1]. However, 10 mM glutamate, which represents a poor nitrogen source in *E. coli*, did not inhibit nitrogenase activity and was used as a positive control. In the absence of the native transcriptional regulatory systems, the T7-dependent *nif* cassette gave rise to substantial nitrogenase activity when cultures were grown in the presence of ammonium or glutamine in comparison with cells grown with glutamate (Figure 5, compare panel B with panel A). However, although nitrogen regulation was bypassed, we observed ∼2 fold reduction in activity in the presence of glutamine and ∼3–4 fold reduction in activity in the presence of ammonium (Figure 5). As the NifL-NifA regulatory system, and the target σ^54^-dependent promoters and UAS sequences are absent from T7 *nif* cassette, this residual response to fixed nitrogen is unexpected. As a further control to examine whether the NifL or NifA proteins could influence activity in the absence of cognate DNA target sites, we prepared constructs in which the *nifLA* operon was reintroduced into the T7 *nif* expression cassette, expressed either from the native *nifL* promoter (pKU7181) or the wild-type T7 promoter (pKU7380). The level of activity in each case in the presence of ammonium was similar to that exhibited by the T7 *nif* cassette lacking *nifL* and *nifA* (Figure S1), demonstrating that the Nif specific regulatory proteins cannot exert nitrogen regulation in the absence of σ^54^ -specific regulatory targets.

We also determined nitrogenase activities of constructs with different T7 promoter strengths with 10 mM ammonium present in the medium. In comparison with cultures grown with 10 mM glutamate, they exhibited a similar ∼3–4 fold reduction in all cases (Figure S2).

Taken together, our results suggest that an alternative mechanism (other than the known transcriptional regulatory circuits) may exist for modulating the system output in relation to the nitrogen source.

### Oxygen availability does not inhibit *nifH* gene expression with the T7-dependent *nif* system

**Figure 6:**
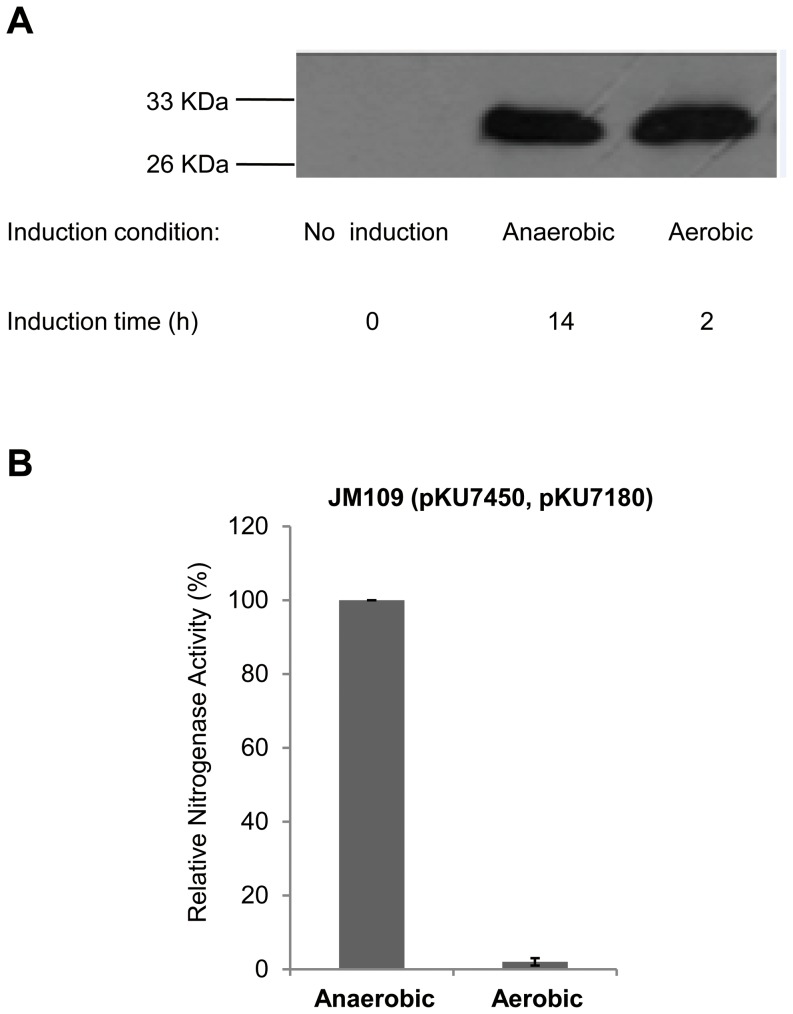
Influence of oxygen on *nifH* gene expression and nitrogenase activities of *E.* *coli* JM109 strain carrying the T7-dependent **** **** ***nif*** **system.** **** (**A**) Western blot analysis of *E. coli* JM109 strain carrying the T7-dependent *nif* system using antiserum against Fe protein (NifH); (**B**) relative nitrogenase activities of *E. coli* JM109 strain under aerobic- and anaerobic- inductions, and 0.2 mM IPTG was used for induction.

In *K. pneumoniae*, *nifLA* expression is oxygen sensitive [22] and transcription from all other *nif* promoters is repressed by oxygen, because NifL inhibits the activity of the NifA transcriptional activator in the presence of oxygen [2], [3]. To test the influence of oxygen on *nif* gene expression of the T7-dependent *nif* system, cells were grown aerobically and induced with IPTG under aerobic conditions. Western blotting with antibody raised against nitrogenase Fe protein indicated that the amount of NifH expressed was similar under both anaerobic and aerobic conditions, either 2 hr or 14 hr post induction (Figure 6A). Therefore, expression of *nifH* is independent of oxygen in the T7-dependent system as expected. However, since the nitrogenase enzyme is extremely oxygen sensitive and irreversibly damaged by O_2_ [2], nitrogenase activity was not detected in the presence of oxygen (Figure 6B).

### Response of the redesigned *nif* system to temperature

**Figure 7:**
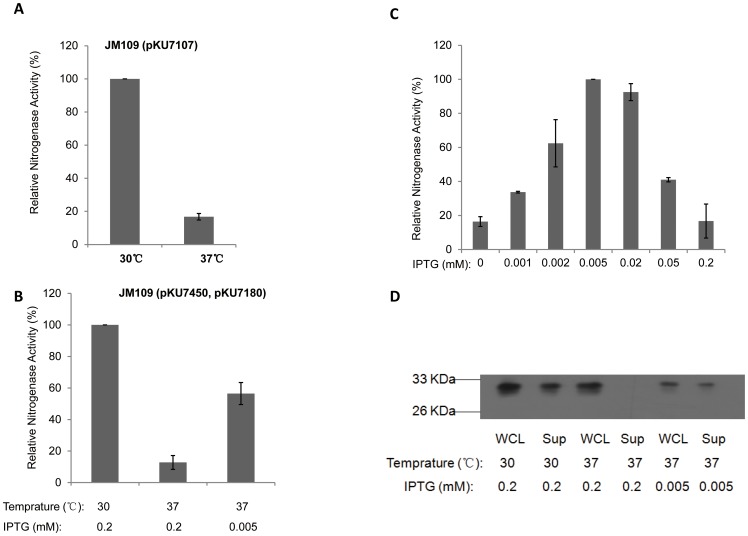
Influence of temperature on the σ^54^-, and T7-dependent *nif* systems. (**A**) Relative nitrogenase activity of *E. coli* JM109 strain carrying the σ^54^-dependent *nif* system at 30°C and 37°C; (**B**), relative nitrogenase activity of *E. coli* strains at 30°C (0.2 mM IPTG induction) and 37°C (either 0.005 mM, or 0.2 mM IPTG as indicated); (**C**), relative nitrogenase activity of *E. coli* JM109 strain carrying the T7-dependent *nif* system in response to various IPTG concentrations at 37°C; (**D**), western blot analysis with antiserum against Fe protein (NifH): WCL (whole cell lysate); Sup (supernatant).

The expression of *nif* operons is repressed at high temperature, due to the temperature sensitive nature of the NifA activator, although the activity of nitrogenase is not oxygen sensitive [23]. Consistent with previous data, very low nitrogenase activity was observed at 37°C with the *E. coli* strain carrying the σ^54^-dependent *nif* system (∼15% activity with respect to that at 30°C, Figure 7A). When the T7-dependent *nif* system was induced with 0.2 mM IPTG at 37°C, nitrogenase activity decreased to ∼20% of the activity observed at 30°C (Figure 7B). We observed that the optimal IPTG concentration for activity at 37°C was 0.005 mM (Figure 7C), representing 60% of the activity observed with 0.2 mM IPTG at 30°C (Figure 7B). The IPTG response curve at 37°C implies that overexpression of Nif polypeptides leads to inhibition of nitrogenase activity at this temperature. To investigate this possibility, we measured the level of NifH protein expression in response to temperature and inducer concentration (Figure 7D). Results showed that, when induced with 0.2 mM IPTG at 37°C, *nifH* expression was not influenced (NifH protein can be detected in the whole cell lysate). However, NifH apparently failed to fold properly, since no protein was evident in the supernatant after sonication and centrifugation of the cells (Figure 7D, compare lanes 3 and 4). Therefore, although the T7 *nif* system bypassed the temperature sensitivity of the NifA activator, protein folding represents another barrier to nitrogen fixation at 37°C, particularly at high inducer concentrations.

## Discussion

It is well documented that *K. pneumoniae nif* gene transcription is stringently regulated in response to fixed nitrogen and oxygen by a complex regulatory cascade that ultimately controls the ability of NifA to activate the nitrogen fixation genes through the upstream activator sequences (UAS) present in their promoters [24]. To examine whether an engineered system can bypass this complex control circuit, we designed a modular *nif* cassette in which transcription of the *nif* operons is driven by T7 RNA polymerase specific promoters and terminators. In this redesigned system, the native NifL and NifA regulatory proteins and the NifA UAS target sequences were removed. This should ablate the currently known mechanisms for transcriptional regulation in response to oxygen and fixed nitrogen. Accordingly, the T7-dependent *nif* system successfully bypasses oxygen regulation of *nif* transcription mediated by the NifL-NifA regulatory system (Figure 6). However, due to the exceptional sensitivity of nitrogenase itself [22], oxygen remains a physical barrier for nitrogen fixation.

Our results demonstrate that the redesigned system is largely independent of controls exerted by the nitrogen regulatory NtrBC system. Nevertheless, some response to the fixed nitrogen source, particularly ammonium, is retained (Figure 5). Potentially, ammonium could influence expression at the post-transcriptional level, or for example, influence protein modification. Although post-translational modification of nitrogenase has not been detected in enteric bacteria in the absence of a functional DraT enzyme [25], covalent modification of the flavodoxin encoded by *nifF* has been demonstrated [22]. However, alternative physiological explanations are possible, for example, effects on the adenylate energy charge or decreases in membrane potential resulting from high levels of external ammonium [26], and consequent generation of the proton motive force [27].

*K. pneumoniae*, NifA is temperature sensitive and consequently the expression of *nif* operons is not activated at high temperatures [23]. Although the T7-dependent *nif* system bypasses this NifA-related regulatory barrier, we observed that under highly induced conditions, the NifH protein becomes insoluble at elevated temperature and consequently only low levels of nitrogenase activity can be detected. Since this protein-folding problem can be overcome to a certain extent by lowering the level of inducer, it would appear that high temperature creates a kinetic barrier towards the appropriate assembly of nitrogenase Fe protein. It may be possible to overcome this newly identified limitation by increasing the expression of *nifM*, which encodes a peptidyl-prolyl cis/trans isomerase required for correct folding of the NifH polypeptide [28], [29].

To evaluate the robustness of the *nif* expression cassette in this study, we replaced the optimal T7 promoter for each modulon with two other T7 promoter variants. Most replacements led to decreased levels of nitrogenase activity indicating that the stoichiometry of *nif* gene expression is very important for nitrogenase assembly and activity. Clearly, the optimal combination of variant T7 promoters employed here provides an appropriate mimic of the native system, as the redesigned *nif* cassette has similar activity to that of the *K. pneumoniae nif* gene cluster. This provides an interesting contrast to a recent study in which the native cluster was completely refactored to remove all non-coding and internal regulatory sequences and replaced with recoded synthetic parts expressed from T7 promoters as three synthetic *nif* operons. However, it is perhaps not surprising that this level of engineering resulted in reduced output and the completely refactored system recovered only around 7% of wild-type nitrogenase activity [30]. In comparison, by keeping the *nif* operons intact and replacing only transcription initiation and termination signals, we have constructed a much simpler T7-dependent system that nevertheless is mainly independent of the native regulatory signals. The complexity of the *nif* gene cluster and the necessity to maintain the stoichiometry of protein expression presents a formidable challenge when completing re-designing the *nif* system from the bottom-up [30]. Organizing genes into artificial operons and controlling expression with synthetic RBS sequences may result in non-optimal protein ratios and hence reduced levels of nitrogenase activity. In retaining the native translation initiation signals and operon structure, we have not encountered these problems, although our artificial system has the disadvantage that is not designed to remove internal regulation. Even so, the residual response to fixed nitrogen is retained in both synthetic systems and is likely to be encoded outside the *nif* cluster itself. Redesigning clusters in this way may provide the first step towards further research aimed at introducing the *nif* genes into eukaryotic organelles for potential application in agro-biotechnology.

## Supporting Information

Figure S1 **Influence of** ***nifL*** **and** ***nifA*** **on nitrogenase activity expressed by the T7 dependent** ***nif*** **system.** Relative nitrogenase activities of *E. coli* JM109 strains carrying (**A**), the T7 dependent *nif* system (pKU7450, pKU7180); (**B**), the T7 dependent *nif* system including the *nifLA* operon driven by the T7 promoter (pKU7450, pKU7380); (**C**), the T7 dependent *nif* system including the *nifLA* operon driven by the native σ^54^-dependent promoter (pKU7450, pKU7181). Activities were measured with cultures grown with 10 mM glutamate (black bars) or 10 mM ammonium (gray bars) after induction with 0.2 mM IPTG. Each experiment was repeated at least three times, and the error bars represent the standard error.
(TIF)

Figure S2 **Influence of ammonium on the nitrogenase activity of T7 dependent** ***nif*** **cassette constructions.** Nitrogenase activities of constructs with different promoter strengths (see Figure 3) were measured on cultures grown with 10 mM glutamate (black bars) or 10 mM ammonium (gray bars) after induction with 0.2 mM IPTG. The activity of the optimal T7 dependent promoter construct (plasmid pKU7180) in cells grown with 10 mM glutamate represents 100% in each case. Each experiment was repeated at least three times, and the error bars represent standard error.
(TIF)

Table S1 **β-galactosidase activities expressed from** ***K. pneumoniae nif*** **promoters.** β-galactosidase activities are shown as a percentage of *nifHDKTY* promoter activity. (Note that the *nifLA* promoter is not included). Each experiment was repeated at least three times, and the values shown are standard error.
(DOC)
