# Supplementary figures and images for: Using Synthetic Biology to Distinguish and Overcome Regulatory and Functional Barriers Related to Nitrogen Fixation
Source: PLoS One. 2013 Jul 25;8(7):e68677. doi: 10.1371/journal.pone.0068677 (PMC3723869; doi:10.1371/journal.pone.0068677)

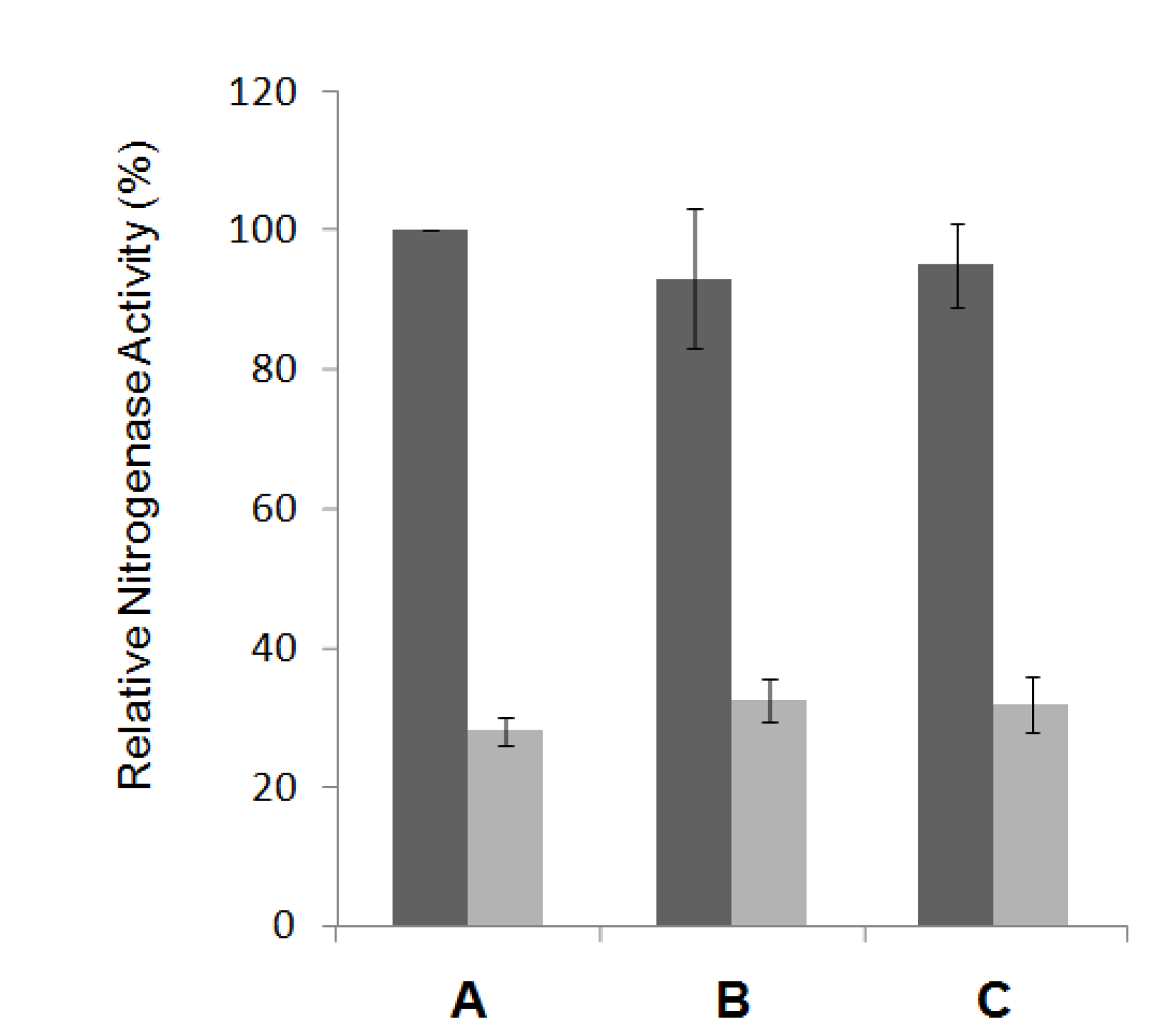

Supplement: Figure S1 — Influence of nifL and nifA on nitrogenase activity expressed by the T7 dependent nif system. Relative nitrogenase activities of E. coli JM109 strains carrying (A), the T7 dependent nif system (pKU7450, pKU7180); (B), the T7 dependent nif system including the nifLA operon driven by the T7 promoter (pKU7450, pKU7380); (C), the T7 dependent nif system including the nifLA operon driven by the native σ54-dependent promoter (pKU7450, pKU7181). Activities were measured with cultures grown with 10 mM glutamate (black bars) or 10 mM ammonium (gray bars) after induction with 0.2 mM IPTG. Each experiment was repeated at least three times, and the error bars represent the standard error. (TIF) [file pone.0068677.s001.tif]

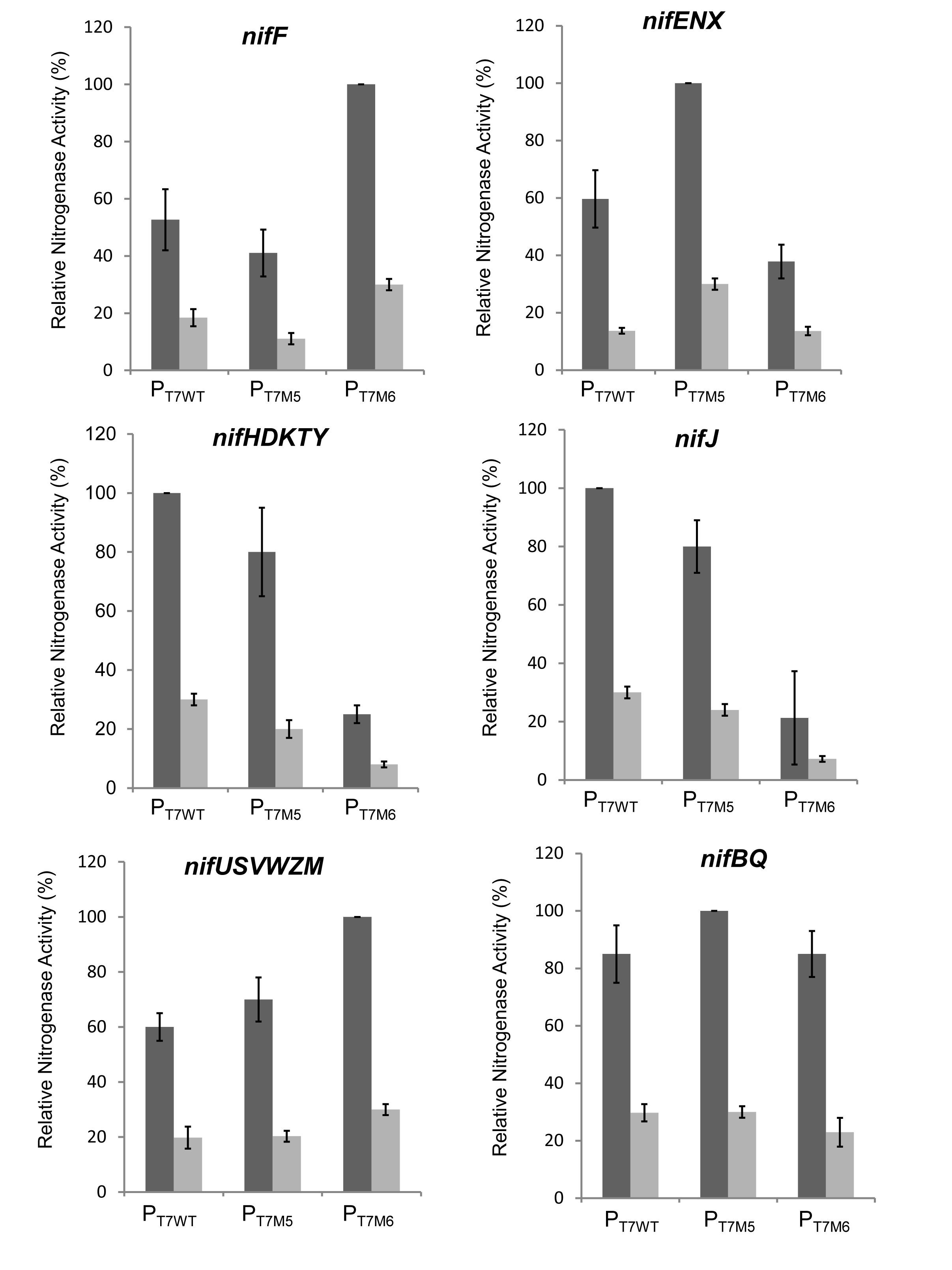

Supplement: Figure S2 — Influence of ammonium on the nitrogenase activity of T7 dependent nif cassette constructions. Nitrogenase activities of constructs with different promoter strengths (see Figure 3) were measured on cultures grown with 10 mM glutamate (black bars) or 10 mM ammonium (gray bars) after induction with 0.2 mM IPTG. The activity of the optimal T7 dependent promoter construct (plasmid pKU7180) in cells grown with 10 mM glutamate represents 100% in each case. Each experiment was repeated at least three times, and the error bars represent standard error. (TIF) [file pone.0068677.s002.tif]
