# Supplementary material for: Using Synthetic Biology to Distinguish and Overcome Regulatory and Functional Barriers Related to Nitrogen Fixation
Source: PLoS One. 2013 Jul 25;8(7):e68677. doi: 10.1371/journal.pone.0068677 (PMC3723869; doi:10.1371/journal.pone.0068677)
Supplement: Table S1 — β-galactosidase activities expressed from K. pneumoniae nif promoters. β-galactosidase activities are shown as a percentage of nifHDKTY promoter activity. (Note that the nifLA promoter is not included). Each experiment was repeated at least three times, and the values shown are standard error. (DOC) [file pone.0068677.s003.doc]

| *nif* promoter | ^1^Relative β-galactosidase activity (%) |
| --- | --- |
| *nifHDKTY*p | 100.0 |
| *nifENX*p | 36.0 ± 3.0 |
| *nifBQ*p | 43.0 ± 5.0 |
| *nifUSVWZM*p | 13.4 ± 0.7 |
| *nifF*p | 18.4 ± 0.8 |
| *nifJ*p | 135.7 ± 1.0 |

1
